# Impact of Psychosocial Factors on Mental Health and Turnover Intention Among Health Workers at Different Occupational Statuses: An Exploratory Cross-Sectional Study in China

**DOI:** 10.3390/ejihpe15050073

**Published:** 2025-05-07

**Authors:** Fuyuan Wang, Min Zhang, Yiming Huang, Yuting Tang, Chuning He, Xinxin Fang, Xuechun Wang, Yiran Zhang

**Affiliations:** School of Population Medicine and Public Health, Chinese Academy of Medical Sciences & Peking Union Medical College, Beijing 100730, China; wangfuyuan@student.pumc.edu.cn (F.W.); huangyiming@sph.pumc.edu.cn (Y.H.); tyting0109@student.pumc.edu.cn (Y.T.); hechuning@student.pumc.edu.cn (C.H.); s2023027004@pumc.edu.cn (X.F.); wangxuechun@student.pumc.edu.cn (X.W.); s2024027025@student.pumc.edu.cn (Y.Z.)

**Keywords:** psychosocial factor, mental health, personnel turnover intention, health personnel, occupational stages

## Abstract

Workplace psychosocial hazards pose significant risks to the well-being of health workers (HWs). This study aimed to explore the levels of psychosocial factors experienced by Chinese and international workers and examine associations between psychosocial factors, health-related outcomes (mental problems and general health), and turnover intention across various occupational stages. A cross-sectional study was conducted using stratified cluster sampling methods at four hospitals in southern China. Psychosocial factors, mental health, general health, and turnover intention were assessed using the Chinese version of the Copenhagen Psychosocial Questionnaire III. Multivariate linear and logistic regression analyses were conducted. A total of 1054 HWs participated in this study (response rate: 80.21%). Compared to international samples, Chinese HWs showed significant differences in nine psychosocial dimensions, particularly interpersonal relations, leadership, and the work–individual interface. Senior hospital managers reported significantly higher stress (51.09 ± 23.88, *p* < 0.001), sleep troubles (53.26 ± 24.92, *p* = 0.003), and poorer general health (57.61 ± 37.26, *p* = 0.035) than other occupational stages. Work–life conflict (*β* = 0.172), emotional demands (*β* = 0.132), and role clarity (*β* = −0.132) were the strongest predictors of mental health issues. Hiding emotions demands (*β* = −0.141) and leadership quality (*β* = 0.130) most strongly predicted general health. The turnover intention rate was 11.01%, with job satisfaction (*OR* = 0.964) being the strongest negative predictor. Reducing psychosocial stressors, particularly in work–life balance, role clarity, emotional demand, and leadership quality, could improve HWs’ well-being and reduce turnover intention.

## 1. Introduction

Psychosocial risk factors in the workplace are generally considered one of the fundamental elements of the working environment, and their impact on the health of the working population has drawn growing concern (Kristensen et al., 2005). The World Health Organization (WHO) defines “psychosocial hazards” as anything in the design or management of work that increases the risk of work-related stress (International Labour Organization, 2022), such as job content design, workload, work pace, organizational culture, work–life balance, and interpersonal relationships at work (International Labour Organization, 2022). In European countries, work-related stress accounts for 50% to 60% of all lost working days, ranking as the second most frequently reported health issue (International Labour Organization, 2014). In the longer term, stress can contribute to a range of physical and mental health issues, including memory impairment, peptic ulcers, inflammatory bowel diseases and musculoskeletal disorders, hypertension, and, ultimately, cardiovascular diseases (International Labour Organization, 2014).

Health workers (HWs) are particularly more vulnerable to psychosocial risks compared to other professions due to the nature of their work, which frequently exposes them to adverse events that can negatively impact their mental health (World Health Organization, n.d.). The Job Demand-Control-Support (JDCS) model posits that high job demands, combined with low job control and insufficient social support, serve as key stressors that detrimentally affect mental health (Nikunlaakso et al., 2022). As one of the most widely recognized frameworks of psychosocial stress in the workplace, the JDCS model assesses psychosocial risks through the Copenhagen Psychosocial Questionnaire (COPSOQ) by combining factors related to job demands (demands at work), job control (work organization and job contents), and social support (interpersonal relations and leadership and social capital). Particularly in healthcare settings, workers frequently face demanding conditions, such as high expectations, long working hours, high job demands, and an imbalance of social support at work (Ruotsalainen et al., 2015). All these factors increase the risk of mental health challenges among health and safety practitioners, including burnout, anxiety, and depression. Moreover, the negative impact on mental health is further exacerbated when workers perceive a lack of control over their work environment, which is common in healthcare settings.

Mental health in the working environment has been consistently rated as a top research and implementation priority by prominent organizations since 2020 (Anger et al., 2024; Kelloway et al., 2023). According to the WHO and the International Labour Organization (ILO), 15% of working-age adults were estimated to have a mental disorder in 2019, and the global economy faces nearly USD 1 trillion annually attributed to the loss of approximately 12 billion workdays due to depression and anxiety (World Health Organization, n.d.). Mental disorders (e.g., depressive disorders, anxiety disorders, and schizophrenia) and mental health problems (e.g., distress, burnout) have substantial implications not only for the affected individuals but also for their employers and the whole of society, prompting many national and international policy and practice initiatives (Rugulies et al., 2023). In September 2022, the WHO released the “Guidelines on Mental Health at Work” and joined by a collaborative policy brief, “Mental Health at Work: Policy Brief,” with the ILO (World Health Organization, 2022b, 2022a), calling on governments and employers to adopt evidence-based interventions in the field of mental health and occupational health for workers. Within the healthcare sector, the consequences of poor mental health are becoming increasingly concerning, particularly given the high-stress environment in which they have been working since the outbreak of the COVID-19 pandemic. The COVID-19 pandemic has significantly intensified this issue, leading to heightened levels of stress, burnout, anxiety, depression, and post-traumatic stress among HWs worldwide (Luo et al., 2020). A number of studies have shown that individuals working in the healthcare system are more likely to experience high levels of workplace stress and long-term mental health issues compared to those in other occupations (Moll, 2014), which in turn gives rise to a higher turnover intention (Hou et al., 2021).

Turnover intention reflects the probability that an organization’s employees will leave voluntarily at some point in the near future (Takase, 2010). It is considered the strongest predictor of actual staff departure, particularly among healthcare professionals (Huang et al., 2023), and is a major factor contributing to staff shortages and undermining healthcare services (Ran et al., 2020). In England, the turnover rate among all general practitioners between 2007 and 2019 increased from 11% to 18.2%, with the proportion of practices experiencing turnover rates between 10% and 40% nearly doubling from 14% in 2009 to 27% in 2019 (Parisi et al., 2021). Similar trends have been observed internationally, with nurse turnover percentages ranging from 15% to 44%, and turnover costs reaching up to USD 48,790 per nurse in Australia (Duffield et al., 2014). According to research in Chinese primary health institutions, turnover intention rates range from 11.80% to 78.40% (Mao et al., 2018). The turnover rates surpass the reasonable threshold of 5–10% (Felicia et al., 2023). Therefore, understanding the factors that contribute to mental health problems and turnover intention in healthcare settings is crucial for improving worker retention and the overall quality of healthcare delivery.

Despite the growing attention to risk factors impacting the well-being and mental health of HWs, only a few studies have been conducted regarding psychosocial aspects. Furthermore, the intersection of psychosocial factors influencing turnover intention remains understudied. There is also a lack of understanding regarding the level of psychosocial factors faced by Chinese HWs, particularly their differential effects across occupational statuses.

To address these gaps, this study aimed to explore the different levels of psychosocial factors experienced by Chinese and international workers (study question 1). We examined the extent of psychosocial factors, health-rated outcomes (mental health problems, and general health), and turnover intention among HWs across different occupational statuses (study question 2). Furthermore, we analyzed the associations between psychosocial factors, health-related outcomes (mental health issues and general health), and turnover intention within this population (study question 3).

To address the study questions and objectives, we propose the following hypotheses:

**Hypothesis 1 (H1).**  *Chinese HWs report distinct psychosocial profiles compared to international samples, with significant disparities in dimensions such as interpersonal relations and work–individual interface.*

**Hypothesis 2 (H2).**  *The occupational stage will significantly affect the levels of psychosocial hazards and mental health, with senior hospital managers reporting higher levels than other occupational groups.*

**Hypothesis 3 (H3).**  *Psychosocial factors will be significant predictors of mental health outcomes, general health, and turnover intention in HWs.*

## 2. Materials and Methods

### 2.1. Study Design and Sampling

This exploratory cross-sectional study used an online version of questionnaires and was conducted at four public hospitals in southern China (hereafter referred to as “the sample hospitals”) between June and July 2023. These four hospitals are part of a medical consortium. The core tertiary public hospital (Hospital A; 850 staff) specializes in infectious diseases. Based on the principle of stratified sampling and recommendations from Hospital A, we selected a secondary public hospital (Hospital B; 380 staff) specializing in psychiatric and infectious diseases, and two primary public hospitals (Hospitals C and D; approximately 200 staff each) focusing on basic public health services and medical care. More information about this tertiary hospital was detailed in our prior research (Huang et al., 2024). This is an example of typical diverse healthcare settings locally. The online questionnaire was distributed to the hospital staff through Wenjuanxing, a widely used web-based survey platform in China. One of the hospital departments facilitated participant recruitment by disseminating the survey link to HWs, including the Infection Control Department and the Hospital Office.

Using stratified cluster sampling methods, we considered the four sample hospitals as four clusters and included all HWs from these hospitals who met our criteria. Inclusion criteria included the following: (a) healthcare professionals with relevant qualifications; (b) voluntary participation with informed consent; (c) regular employees who have been employed for over one year; (d) trainees, interns, or students at the hospital for at least six months. Exclusion criteria included the following: (a) absence from duty for over one month during the investigation period and (b) failure to complete the questionnaire in the opening hours. The hospital departments responsible for recruiting HWs distributed the survey link to qualified participants, and our research team performed rigorous verification of all participants meeting the inclusion and exclusion criteria before data analysis.

A total of 1067 questionnaires were collected, with 13 excluded due to logical errors (such as age less than 10 years or inconsistencies between work years, title, and salary) or missing key values, resulting in a final sample of 1054 questionnaires. The response rate was 80.21%. All participants are Chinese nationals.

Participation was voluntary, and informed consent was presented on the first page of the questionnaire. Comprehensive explanations regarding the study’s attributes, benefits, and applications were provided to all participants. All data were anonymized to ensure the privacy and confidentiality of the participants. Additionally, the participants were assured that their data would be used solely for research purposes.

### 2.2. Measures

#### 2.2.1. Psychosocial Factors

Psychosocial factors were assessed using the Chinese version of the Copenhagen Psychosocial Questionnaire III (COPSOQ III) (Burr et al., 2019). The COPSOQ has been recognized as a valuable instrument by several organizations (International Labour Organization, 2016; Leka & Jain, 2010) and has demonstrated cross-cultural validity, with translations available in over 25 languages and applications in 40 countries worldwide (Burr et al., 2019). The COPSOQ allows for operationalizing the most prominent work environment theories, including the most relevant psychosocial domains, such as job demand–control–social support, effort-rewards, job resources, work–family conflict, and social capital (COPSOQ International Network, 2019). The COPSOQ III was translated into Chinese and culturally adapted. The Chinese version of the COPSOQ III exhibited excellent internal consistency (Cronbach’s α = 0.92), indicating its strong reliability and validity among Chinese workers (Huang et al., 2025).

**Figure 1:**
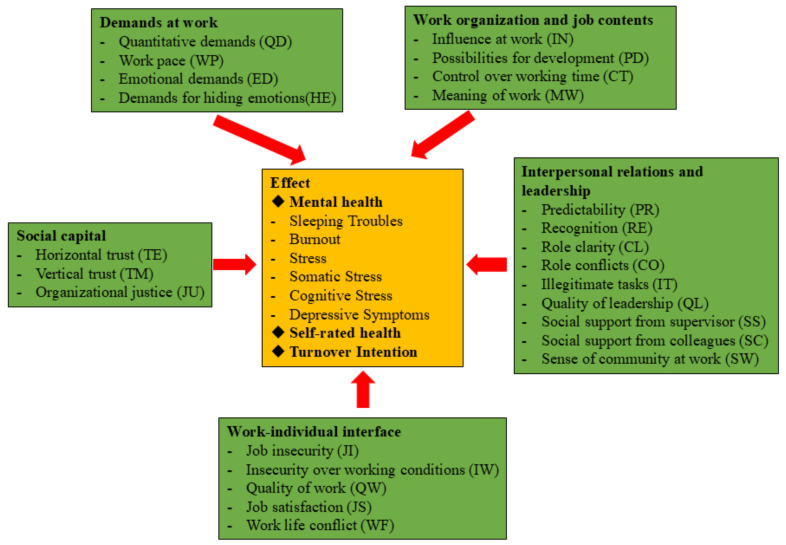
Content of the Chinese version of COPSOQ III in this study.

In this study, we employed the international middle and core version of the COPSOQ III, which consists of 59 items across 25 dimensions, organized into 5 domains: Demands at Work, Work Organization and Job Contents, Interpersonal Relations and Leadership, Work–Individual Interface, and Social Capital (COPSOQ International Network, 2019) (Figure 1). All items were measured with a 5-point Likert scale with the following options: 1 (Never/hardly ever), 2 (Seldom), 3 (Sometimes), 4 (Often), and 5 (Always). All forward and reverse scoring items were transformed to a value ranging from 0 to 100, with higher scores indicating greater expression on the corresponding scale. The reliability of COPSOQ III scales proved to be good, with a Cronbach’s α coefficient of more than 0.7, based on mean values summarized across the five core domains in a prior international validation study (Burr et al., 2019). Additionally, the construct validity was confirmed to be good (Bjorner & Pejtersen, 2010).

#### 2.2.2. Mental Health Issues

The mental health of HWs was measured using COPSOQ III, which consists of 15 items across 4 dimensions: sleeping troubles, burnout, stress, and depressive symptoms. Each item was evaluated using a 5-point Likert scale, yielding a total score ranging from 0 (Never/hardly ever) to 100 (Always). Higher scores indicated a more severe expression of the corresponding mental state. The Cronbach α coefficient for this scale was 0.83.

#### 2.2.3. General Health

General health was measured by a single question of COPSOQ III: “In general, would you say your health is…?” The answers include excellent (100), very good (75), good (50), fair (25), and poor (0). Higher scores indicated a better condition of general health.

#### 2.2.4. Turnover Intention

Turnover intention was measured using a self-report question developed specifically for this study: “Do you intend to resign?” The participants were provided with the response options “yes” and “no”.

### 2.3. Statistical Analyses

Descriptive analysis was employed to describe the demographic and occupational characteristics of the study population, along with their psychosocial characteristics, mental health state, and turnover intention.

To evaluate the observed differences in psychosocial characteristics between HWs and an international reference sample of 23,361 workers from six countries (Burr et al., 2019), we employed a nomenclature commonly used in previous COPSOQ studies (Kersten et al., 2014; Wagner et al., 2020). Specifically, a difference of at least 5 points in the mean values of groups is regarded as a clear difference, and a deviation of 10 or more points is classified as a very clear deviation. This nomenclature is grounded in the effect size measure (Cohen’s α), considering that COPSOQ scales typically have standard deviations ranging from 15 to 25 points; thus, a 5-point difference corresponds to a small to intermediate effect size (0.2–0.33), while a 10-point difference indicates a moderate to strong effect size (0.4–0.66) (Nübling et al., 2010).

One-way analyses of variance were used to verify if there were significant differences in psychosocial factors, mental health diagnoses, and general health of HWs across different occupational statuses (senior management/hospital manager, department manager, department staff, and intern/trainee/student). Pearson’s chi-square test of independence was used to compare turnover intention across career stages. To explore the relationships between health-related outcomes (mental health problems and general health), turnover intention, and the level of psychosocial factors, the Pearson correlation was used. All variables that showed a non-significant correlation (*p* > 0.01) with any dependent variables were excluded from the multivariate analysis. Multivariate linear and logistic regression models were used to explore the association between 5 domains of psychosocial factors (independent variables) and health-related outcomes (mental health problems and general health) (dependent variables) and turnover intention (dependent variables) depending on the data type. Parameter estimates were considered statistically significant if the two-tailed *p*-values were below 0.05. To evaluate the influence of common method bias (CMB), Harman’s single-factor test was conducted. The results revealed 14 factors with a characteristic root >1, among which the first factor accounted for 20.89% of the variance, which suggests that there is no significant common method bias in this study. All data analysis and processing were conducted using R software version 4.3.2 (R Development Core Team, Vienna, Austria).

## 3. Results

### 3.1. Demographic Characteristics of Health Workers

The final sample size was 1054 HWs, with a response rate of 80.21% (1054/1314). Table 1 presents the demographic and occupational characteristics of the study participants categorized by various occupational statuses, including department staff (75.43%), intern/trainee/student (11.48%), department manager (10.91%), and hospital senior manager (2.18%). Female HWs (74.00%) outnumbered their male counterparts. Occupational data showed that a high proportion of nurses and doctors were characteristic of the whole study population (47.44% of nurses and 23.91% of doctors) and each occupational status. Additionally, most participants reported having been employed in a medical institution for a duration of 6 to 10 years (24.00%).

### 3.2. The COPSOQ III: Descriptive Statistics of Dimensions

Table 2 presents the mean values and 95% CIs for all dimensions of psychosocial stressors, as well as a comparison between Chinese participants in our study and a normative sample of 23,361 employees from six countries (study question 1). The mean scores for psychosocial stressors among our participants ranged from 35.57 (95% CI: 34.49−36.66) to 72.02 (95% CI: 70.70−73.34). The significant difference between the two populations primarily focuses on domains of interpersonal relations and leadership and the work–individual interface. The comparison of Chinese HWs with international workers revealed significantly better outcomes in recognition (65.18 vs. 55.00) and insecurity over working conditions (52.54 vs. 41.00) with medium to large effects, and higher levels of job insecurity (46.10 vs. 39.00) and job satisfaction (61.79 vs. 56.00) in a small effect. Additionally, we identified a significantly lower sense of community at work (66.46 vs. 77.00) for Chinese HWs with a medium to large effect size. Chinese HWs also indicated lower role clarity (69.34 vs. 75.00), social support from supervisor (60.09 vs. 68.00), social support from colleagues (63.08 vs. 69.00), and quality of work (65.06 vs. 71.00), all of which exhibited a small effect (study question 1).

### 3.3. The COPSOQ III: General Psychosocial Exposure by Occupational Status

Table 3 shows the psychosocial exposures across the five COPSOQ III domains stratified by occupational status (study question 2). Significant differences between occupational statuses were observed across all dimensions, except for predictability, recognition, social support from supervisors, horizontal trust, and vertical trust. Post hoc comparisons using Tukey’s HSD test demonstrated that department managers exhibited a notably higher level of demands at work (53.95 ± 11.51 vs. 46.36 ± 14.77, *p* = 0.003) and work organization and job contents (54.78 ± 11.36 vs. 49.36 ± 11.76, *p* = 0.043) compared to department staff. However, no significant disparities were observed among the other occupational groups. Regarding the work–individual interface domain, interns/trainees/students displayed a significantly higher level than hospital senior managers (55.52 ± 12.05 vs. 48.81 ± 14.84, *p* = 0.049).

### 3.4. Mental Health and Turnover Intention by Occupational Status

Table 4 presents the mental health status and turnover intention across different occupational statuses (study question 2). The mean values of mental health problems ranged from 26.67 ± 20.50 to 38.15 ± 22.00, suggesting that all HWs experienced mental health issues, with a frequency ranging from sometimes to often. Statistically, differences in occupational status were identified across all mental health problems and general health, except for burnout. Tukey’s post hoc analysis demonstrated that senior hospital managers reported the highest levels of sleeping troubles (53.26 ± 24.92, all *p* < 0.01) and the worst level of general health (57.61 ± 37.26, all *p* < 0.01) compared to other occupational groups. Regarding stress, senior hospital managers also exhibited significantly higher levels than department staff (*p* = 0.001) and interns/trainees/students (*p* = 0.005). In addition, the overall turnover intention among all HWs was 11.01%, with significant differences observed across the four occupational groups. Among these, department staff demonstrated the highest turnover intention, at 6.74%.

### 3.5. Consequences of Psychosocial Exposure

Correlations between health-related outcomes (mental health issues and general health), turnover intention, and all psychosocial factors are presented in Table 5. Significant correlations (*p* < 0.01) were found between all dimensions in the domains of work demands, work–individual interface, and social capital with health-related outcomes and turnover intention. For the remaining dimensions, total mental health issues did not show a significant correlation with control over working time (r = 0.074); general health did not correlate significantly with influence at work (r = 0.04), control over working time (r = 0.077), meaning of work (r = 0.062), and role conflicts (r = 0.07); and turnover intention did not correlate significantly with possibilities for development (r = 0.076), predictability (r = 0.058), quality of leadership (r = 0.064), and social support from colleagues (r = 0.075). Consequently, these non-significant variables were excluded from the subsequent multivariate regression analysis.

**Table 5:** Relationship between psychosocial factors and mental health issues, general health, and turnover intention.

Domain	Dimension	Sleeping	Burnout	Stress	Depressive	Total Mental	General Health	Turnover Intention
Demands at Work	QD	0.274 ***	0.388 ***	0.369 ***	0.384 ***	0.404 ***	−0.186 ***	0.294 ***
	WP	0.265 ***	0.344 ***	0.303 ***	0.207 ***	0.321 ***	−0.213 ***	0.175 ***
	ED	0.333 ***	0.445 ***	0.429 ***	0.385 ***	0.455 ***	−0.194 ***	0.286 ***
	HE	0.294 ***	0.381 ***	0.328 ***	0.253 ***	0.360 ***	−0.199 ***	0.221 ***
Work Organization and Job Contents	IN	0.107 ***	0.115 ***	0.137 ***	0.153 ***	0.146 ***	0.040	0.089 **
	PD	−0.124 ***	−0.160 ***	−0.157 ***	−0.146 ***	−0.168 ***	0.165 ***	−0.076 *
	CT	0.021	0.028	0.047	0.167 ***	0.074 *	0.077 *	0.099 **
	MW	−0.111 ***	−0.155 ***	−0.177 ***	−0.236 ***	−0.193 ***	0.062 *	−0.126 ***
Interpersonal Relations and Leadership	PR	−0.095 **	−0.131 ***	−0.101 **	−0.060	−0.111 ***	0.153 ***	−0.058
	RE	−0.136 ***	−0.205 ***	−0.212 ***	−0.194 ***	−0.213 ***	0.167 ***	−0.139 ***
	CL	−0.157 ***	−0.219 ***	−0.225 ***	−0.280 ***	−0.251 ***	0.145 ***	−0.148 ***
	CO	0.206 ***	0.275 ***	0.275 ***	0.219 ***	0.279 ***	−0.070 *	0.139 ***
	IT	0.238 ***	0.347 ***	0.318 ***	0.247 ***	0.329 ***	−0.115 ***	0.199 ***
	QL	−0.127 ***	−0.159 ***	−0.172 ***	−0.164 ***	−0.178 ***	0.215 ***	−0.064 *
	SS	−0.101 **	−0.162 ***	−0.185 ***	−0.129 ***	−0.164 ***	0.191 ***	−0.137 ***
	SC	−0.088 **	−0.131 ***	−0.150 ***	−0.176 ***	−0.155 ***	0.125 ***	−0.075 *
	SW	−0.158 ***	−0.206 ***	−0.229 ***	−0.262 ***	−0.244 ***	0.167 ***	−0.111 ***
Work–Individual Interface	JI	0.218 ***	0.252 ***	0.264 ***	0.282 ***	0.291 ***	−0.121 ***	0.105 **
	IW	0.273 ***	0.349 ***	0.345 ***	0.290 ***	0.360 ***	−0.147 ***	0.166 ***
	QW	−0.095 **	−0.122 ***	−0.142 ***	−0.171 ***	−0.151 ***	0.156 ***	−0.085 **
	JS	−0.186 ***	−0.287 ***	−0.273 ***	−0.231 ***	−0.279 ***	0.235 ***	−0.220 ***
	WF	0.359 ***	0.498 ***	0.456 ***	0.412 ***	0.493 ***	−0.218 ***	0.235 ***
Social Capital	TE	−0.106 **	−0.169 ***	−0.189 ***	−0.164 ***	−0.179 ***	0.096 ***	−0.096 ***
	TM	−0.131 ***	−0.188 ***	−0.206 ***	−0.175 ***	−0.199 ***	0.146 ***	−0.118 ***
	JU	−0.159 ***	−0.239 ***	−0.260 ***	−0.210 ***	−0.248 ***	0.191 ***	−0.108 ***

**Figure 2:**
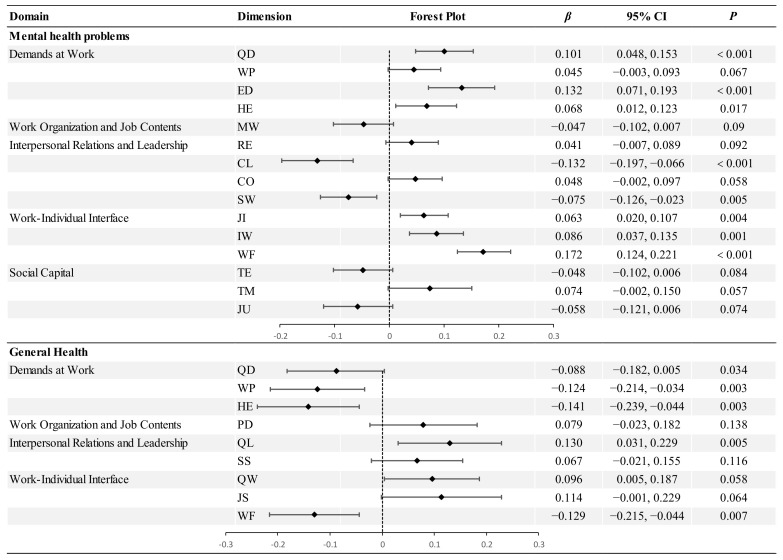
Results of multivariable linear regression analysis of the relationship between psychosocial factors and mental health problems and general health (N = 1054).

**Figure 3:**
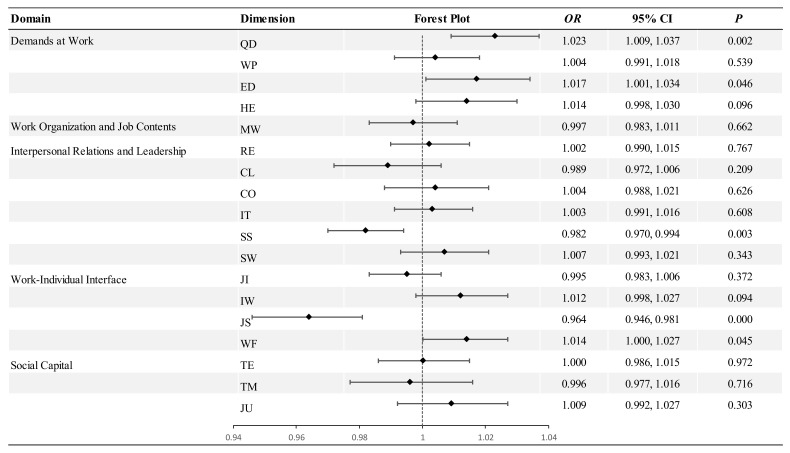
Results of logistic regression analysis of the relationship between psychosocial factors and turnover intention (N = 1054).

* *p* < 0.05; ** *p* < 0.01; *** *p* < 0.001. The consequences of psychosocial factors, including health-related outcomes (mental health issues and general health) and turnover intention, are presented in Figure 2 and Figure 3 (study question 3).

#### 3.5.1. Mental Health Issues

Figure 2 shows the results of multivariable regression models examining the relationship between psychosocial exposure and mental health issues (study question 3). After controlling for key covariates, mental health issues were most strongly predicted by work–life conflict (*β* = 0.172, 95% CI: 0.124–0.221) and emotional demands (*β* = 0.132, 95% CI: 0.071−0.193), both of which had positive associations. In contrast, role clarity (*β* = −0.132, 95% CI: −0.197–−0.066) was negatively associated with mental health issues. The total model was significant (*p* < 0.001) and accounted for 40.47% of the variance in mental health issues (adjusted R^2^).

#### 3.5.2. General Health

As shown in Figure 2, the multivariable regression models revealed that the general health of HWs was significantly predicted by hiding emotional demands (*β* = −0.141, 95% CI: −0.239–−0.044) and quality of leadership (*β* = 0.130, 95% CI: 0.031−0.229) (study question 3). The total model was statistically significant (*p* < 0.001) and explained 14.7% of the variance in general health (adjusted R^2^).

#### 3.5.3. Turnover Intention

Figure 3 illustrates the results of the multivariable logistic regression models examining the relationship between psychosocial exposure and turnover intention among HWs (study question 3). After adjusting for all key covariates, the models revealed that job satisfaction (*OR* = 0.964, 95% CI: 0.946–0.981) demonstrated the strongest negative association with turnover intention, while quantitative demands (*OR* = 1.023, 95% CI: 1.009–1.037) exhibited the strongest positive association with turnover intention.

## 4. Discussion

This study identifies the impact of psychosocial factors on mental health, general health, and turnover intention in four hospital settings in southern China. The results provide new insights into the psychosocial hazards encountered by Chinese HWs at different occupational statuses, particularly highlighting the distinct differences that may be unique to China compared to international samples. Consistent with our hypotheses, the study identifies critical psychosocial factors that significantly influence health-rated outcomes and turnover intention, offering important insights into the specific challenges faced by Chinese HWs and suggesting potential areas for targeted intervention to improve well-being and reduce turnover in this high-stress profession. Therefore, our research findings validate all of the hypotheses posited.

### 4.1. Chinese and International HWs Experienced Different Psychosocial Stressors

The results of our study indicated substantial differences in psychosocial stressors between Chinese HWs and the international reference sample drawn from six countries (Burr et al., 2019). These differences were particularly pronounced in the domains of interpersonal relations and leadership and the work–individual interface. Specifically, the Chinese group reported significantly more adverse values across most dimensions, such as lower levels of social support from supervisors and colleagues, reduced sense of community at work, poorer role clarity, and higher levels of job insecurity over working conditions. These findings suggest a more challenging psychosocial work environment for Chinese HWs compared to their international counterparts.

Interestingly, despite these increased stressors, Chinese HWs reported higher levels of job satisfaction and recognition compared to international HWs. This discrepancy suggests a more complex interaction between stressors at work and cultural values. One explanation for this phenomenon may be attributed to the Confucian-based work ethic that permeates many aspects of Chinese society and workplace culture. Confucian cultural values emphasize collectivism, endurance, hard work, and the importance of personal networks (*guanxi*). Such values may shape workers’ attitudes toward their jobs, encouraging them to devote themselves fully to their jobs and take full responsibility, leading to a strong sense of loyalty and commitment to their work organizations (Lu et al., 2011). According to Confucian principles, the duty to contribute to a better community and maintain harmony in the workplace is paramount, which may lead Chinese HWs to persist in their roles despite difficult working conditions. The emphasis on endurance and commitment when facing adversity may mitigate some of the negative effects of stress, helping workers feel more satisfied with their jobs, even when the work environment is challenging.

This aligns with findings from Lu et al., who suggest that Chinese work values can mitigate the negative effects of work stress on employee well-being (Lu et al., 2011). However, this result contrasts with previous research indicating that Chinese workers generally report lower levels of job satisfaction compared to workers in 35 other countries (Zhang et al., 2019). This study attributed the discrepancy to different job attributes and unmet expectations, particularly regarding job expectations for interesting work, high pay, and opportunities for advancement. It is important to note, however, that our study focused exclusively on medical workers in a single Chinese province. Therefore, the inconsistency between these results warrants further investigation. Further study should focus on the roles of cultural values, organizational structures, and policy interventions in shaping the psychosocial well-being of HWs.

### 4.2. Associations Between Psychosocial Factors and Health-Rated Outcomes Across Occupational Statuses

Our study reveals that HWs in China experience a variety of mental health issues at different stages of their careers, with senior hospital managers reporting significantly higher levels of stress and sleep disturbances and poorer levels of general health compared to other occupational groups. Prior research has pointed out that a higher occupational status often entails greater responsibility and more pressure (Li et al., 2016; Shanafelt et al., 2012). On one hand, this could be compounded by the high levels of responsibility and decision-making in leadership roles, which are associated with elevated stress levels on themselves (Siegrist, 1996). On the other hand, supervisor support roles play a key role in managing these stressors. Previous studies have emphasized the importance of supervisor support in navigating complexity, particularly in ensuring the delivery of high-quality patient care during and after COVID-19 (Hebles et al., 2021; Tevis et al., 2022; Walsh & Kabat-Farr, 2022). This is not unique to the healthcare sector; similar patterns have been observed in other industries, including hospitality, academia, and remote work (Charoensukmongkol & Phungsoonthorn, 2021; Kikunaga et al., 2023; Tu et al., 2021).

However, the reality is that leaders of healthcare institutions are required to both implement strategies communicated by their superiors and provide support to their subordinates during challenging times, and they are equally affected by the ongoing crisis of COVID-19, further exacerbating their psychosocial stressors and mental health challenges (Spanos et al., 2024). These dynamics may contribute to the heightened psychological strain observed among healthcare managers in our study, such as the domain of work demand, work organization, and job contents, highlighting the critical need for targeted interventions to support their mental well-being.

The multivariable regression analyses of the study revealed that specific psychosocial factors, such as work–life conflict, emotional demands, and role clarity, had the strongest associations with mental well-being after controlling for key covariates. According to the JDCS model, high job demands coupled with low job control can lead to increased stress, which in turn can exacerbate work–life conflict (Nikunlaakso et al., 2022). This conflict not only affects employees’ job performance but also leads to mental health issues such as anxiety, depression, and job burnout (Liu & Hong, 2023). These findings are consistent with the broader literature, which highlights that conflicts between professional and personal life contribute to psychological distress and burnout (Dyrbye et al., 2019), even bringing USD 24 billion in incremental health expenditures (Goh et al., 2016). In line with the results of the study in the post-COVID era, work–life conflict still played a vital role in determining workers’ job satisfaction (Al Dilby & Farmanesh, 2023). In addition to direct effects, Wang et al. showed that work–life conflict indirectly affects mental health by influencing HWs’ job satisfaction and work engagement (Yang et al., 2024). Considering the nature of their work, physicians frequently face demanding schedules, including extended hours, overtime, and night shifts, which often result in irregular working hours. These factors make it challenging for them to spend quality time with their families. In addition, the high workload and intense work pressure are pervasive issues for HWs, who are tasked with managing large numbers of patients while also navigating the risks of medical malpractice and the complexities of strained doctor–patient relationships. This constant pressure creates an environment where relaxation and recovery after work become difficult, thereby negatively impacting their family life and overall well-being. These findings underscore the importance of considering both work-related stressors and personal life challenges when designing interventions to promote mental health among HWs.

### 4.3. Effect of Psychosocial Stressors on Turnover Intention

Turnover intention among HWs is a critical issue globally, and our study found a total prevalence rate of 11.01% in four Chinese hospitals, with notable differences across occupational statuses. Department staff were found to have the highest turnover intention, which may reflect dissatisfaction with job demands, leadership responsibilities, and a perceived lack of organizational support. Job satisfaction emerged as the strongest negative predictor of turnover intention in this study, consistent with a large body of research highlighting the centrality of job satisfaction in influencing retention rates in healthcare (McVicar, 2003). The results suggest that addressing factors that enhance job satisfaction, such as leadership support, organizational culture, and career development opportunities, may be effective strategies for reducing turnover intention across different career stages. This aligns with recommendations from previous studies advocating for workplace interventions that focus on enhancing the work environment to improve job satisfaction and reduce the risk of turnover (Duan et al., 2019).

### 4.4. Intervention Recommendations for Addressing Psychosocial Factors

Given the significant associations between psychosocial factors and health outcomes, as well as the impact on turnover intention, we suggest the following recommendations for different aspects.

Interventions for senior hospital managers. Given the elevated stress and sleep disturbances and reduced general health conditions among senior managers, hospitals should provide corresponding training programs that focus on stress management, decision-making skills, and time management. Besides, they should lighten administrative burdens on senior leaders by hiring support staff or providing more vacations to improve work–life balance.

Supporting work–life balance. Implement flexible work scheduling systems, such as adjustable shift arrangements, to help HWs better balance work and personal lives. Also, provide childcare facilities or partner with local childcare services to relieve the childcare burden of HWs with young children, enabling them to focus more on work.

Enhancing emotional demand and social support systems. Establish confidential counseling services, staffed by professional psychologists or counselors, where HWs can process high-stress incidents. These professionals can offer individual counseling, group therapy, or stress-management workshops for HWs. Additionally, create a supportive work culture that encourages open communication about emotions.

Promoting job satisfaction to reduce turnover. HR departments should create transparent promotion criteria and provide continuing education to foster professional growth. Additionally, creating a more inclusive and supportive work environment will help retain staff by fostering a sense of belonging and commitment.

Hospitals should implement systematic interventions, such as occupational health and safety management systems, to address these questions comprehensively. Policymakers should consider enacting regulations that mandate better working and psychosocial conditions for HWs.

### 4.5. Strengths and Limitations

The results provide valuable insights into the psychosocial factors affecting HWs, an area of growing concern in the context of heavy medical demand. However, the level of awareness and the impact of these psychosocial factors among workers in China, compared to international standards, remain underexplored. By addressing this gap, the study offers an important reference for future research. Besides, to the best of our knowledge, this investigation represents one of the first to use COPSOQ III in Chinese hospital settings across occupational levels. We focused on HWs at various career stages, allowing for a comprehensive understanding of how psychosocial factors affect different groups within the workforce. Furthermore, the research explores the effects of these factors on health, mental health, and turnover intention, providing a solid evidence base for the implementation of proactive measures. Additionally, the inclusion of a large, diverse sample from multiple medical consortium hospitals in southern China enhances the generalizability of the findings to similar healthcare settings in China.

However, several limitations should be acknowledged. First, the cross-sectional design limits the ability to establish causal relationships between psychosocial factors, mental health, and turnover intention. Longitudinal studies are needed to examine the temporal dynamics of these associations and to better understand changes in psychosocial stressors over time. Additionally, the study does not explore the gender-based, organizational, or cultural factors specific to Chinese healthcare institutions, which may further influence HWs’ psychosocial experiences. This underscores the need for future qualitative research. Third, all data were collected through self-report questionnaires, which are inherently subject to recall bias, social desirability bias, and selection bias, particularly when focusing on psychological questions. Although the data were collected anonymously, the participants still feared potential punishment or retaliation for reporting sensitive items, such as turnover intention, leading to an underreported rate. Finally, previous studies have indicated that occupation-based surveys may be prone to response biases, which could lead to higher reported rates of psychological distress compared to those observed in population-based surveys (Goodwin et al., 2013). This discrepancy is often attributed to the tendency of employees to express dissatisfaction or frustration with their current work situation, whether consciously or unconsciously (Goodwin et al., 2013). Such biases are thought to reflect the emotional toll of work-related stress, which may not be fully representative of the broader population’s experiences. Therefore, caution should be exercised when interpreting findings from occupation-based surveys.

## 5. Conclusions

This study highlights the significant role of workplace psychosocial factors in shaping the health, mental well-being, and turnover intention of HWs in China. Our findings reveal that Chinese HWs encounter distinct psychosocial stressors compared to their international counterparts. Senior hospital managers were identified as a key group requiring focused attention, as they exhibited significantly higher levels of mental health issues and poorer general health. These results highlight the urgent need for targeted interventions to mitigate psychosocial stressors in healthcare environments, particularly those related to work–life conflict, emotional demands, and leadership quality, which contribute to the deteriorating both mental and general health of HWs. Additionally, interventions aimed at enhancing job satisfaction could reduce turnover intention, thereby promoting the long-term resilience of the healthcare system. The implications of these findings are far-reaching, extending to the sustainability of the healthcare system and workforce stability, especially in the post-pandemic era. By addressing these psychosocial challenges, healthcare institutions can foster a healthier, more engaged workforce, ultimately enhancing patient care quality and operational efficiency. These insights provide a foundation for evidence-based policies to support the well-being of healthcare professionals and ensure the sustainability of healthcare systems globally. Future research should explore the long-term effects of psychosocial stress among health professions, including gender-based disparities, and expand analyses to include a broader range of high-stress professionals, such as emergency services, social workers, and education. Additionally, research should evaluate effective evidence-based interventions to mitigate psychosocial stress, including organizational-, social-, and individual-level approaches.

## Figures and Tables

**Table 1:** Demographic characteristics of health workers across four occupational statutes (N = 1054).

		Total (N = 1054)	Senior Manager/ Hospital Manager (n = 23)	Department Manager (n = 115)	Department Staff (n = 795)	Intern/ Trainee/Student (n = 121)
Gender	Female	780 (74.00)	9 (39.13)	72 (62.61)	624 (78.49)	75 (61.98)
	Male	274 (26.00)	14 (60.87)	43 (37.39)	171 (21.51)	46 (38.02)
Age	<25	211 (20.02)	2 (8.70)	23 (20.00)	185 (23.27)	1 (0.83)
	25~	415 (39.37)	6 (26.09)	52 (45.22)	343 (43.14)	14 (11.57)
	35~	291 (27.61)	7 (30.43)	26 (22.61)	200 (25.16)	58 (47.93)
	45~	113 (10.72)	7 (30.43)	13 (11.30)	58 (7.30)	35 (28.93)
	55~	24 (2.28)	1 (4.35)	1 (0.87)	9 (1.13)	13 (10.74)
Education	High school and below	58 (5.50)	3 (13.04)	4 (3.48)	36 (4.53)	15 (12.40)
	Junior college	264 (25.05)	2 (8.70)	18 (15.65)	205 (25.79)	39 (32.23)
	Undergraduate	676 (64.14)	12 (52.17)	74 (64.35)	526 (66.16)	64 (52.89)
	Postgraduate	56 (5.31)	6 (26.09)	19 (16.52)	28 (3.52)	3 (2.48)
Occupation	Doctor	252 (23.91)	9 (39.13)	36 (31.30)	181 (22.77)	26 (21.49)
	Nurse	500 (47.44)	6 (26.09)	34 (29.57)	428 (53.84)	32 (26.45)
	Technical support	125 (11.86)	1 (4.35)	13 (11.30)	92 (11.57)	19 (15.70)
	Administrative staff	177 (16.79)	7 (30.43)	32 (27.83)	94 (11.82)	44 (36.36)
Job title	Senior professional	165 (15.65)	11 (47.83)	51 (44.35)	99 (12.45)	4 (3.31)
	Intermediate professional	340 (32.26)	6 (26.09)	39 (33.91)	288 (36.23)	7 (5.79)
	Junior professional	440 (41.75)	6 (26.09)	20 (17.39)	365 (45.91)	49 (40.50)
	None/assistant	109 (10.34)	0 (0.00)	5 (4.35)	43 (5.41)	61 (50.41)
Working years	<1	146 (13.85)	5 (21.74)	5 (4.35)	35 (4.40)	101 (83.47)
	1~	236 (22.39)	4 (17.39)	15 (13.04)	203 (25.53)	14 (11.57)
	6~	253 (24.00)	2 (8.70)	16 (13.91)	233 (29.31)	2 (1.65)
	11~	184 (17.46)	5 (21.74)	32 (27.83)	144 (18.11)	3 (2.48)
	15~	235 (22.30)	7 (30.43)	47 (40.87)	180 (22.64)	1 (0.83)
Night work	Yes	620 (58.82)	6 (26.09)	40 (34.78)	522 (65.66)	52 (42.98)
	No	434 (41.18)	17 (73.91)	75 (65.22)	273 (34.34)	69 (57.02)
Shift work	Yes	761 (72.20)	10 (43.48)	53 (46.09)	616 (77.48)	82 (67.77)
	No	293 (27.80)	13 (56.52)	62 (53.91)	179 (22.52)	39 (32.23)

Data are n (%).

**Table 2:** Comparison of psychosocial factors between Chinese health workers and international workers based on COPSOQ III dimensions.

Domain	Dimension	Abbreviation	Mean (Chinese Workers)	95% CIs	Mean (International Workers ^a^)	Difference (Chinese vs. International)
Demands at work	Quantitative demands	QD	37.02	35.94−38.09	39.00	−1.98
	Work pace	WP	58.54	57.22−59.86	61.00	−2.46
	Emotional demands	ED	43.52	42.36−44.67	47.00	−3.48
	Demands for hiding emotions	HE	54.12	52.92−55.32	57.00	−2.88
Work organization and job contents	Influence at work	IN	45.42	44.39−46.45	42.00	3.42
	Possibilities for development	PD	62.27	61.11−63.43	66.00	−3.73
	Control over working time	CT	35.57	34.49−36.66	39.00	−3.43
	Meaning of work	MW	72.02	70.70−73.34	72.00	0.02
Interpersonal relations and leadership	Predictability	PR	57.70	56.43−58.97	56.00	1.70
	Recognition	RE	65.18	63.78−66.58	55.00	10.18 **
	Role clarity	CL	69.34	68.15−70.53	75.00	−5.66 *
	Role conflicts	CO	47.72	46.55−48.89	45.00	2.72
	Illegitimate tasks	IT	45.87	44.47−47.28	43.00	2.87
	Quality of leadership	QL	57.47	56.23−58.71	61.00	−3.53
	Social support from supervisor	SS	60.09	58.76−61.42	68.00	−7.91 *
	Social support from colleagues	SC	63.08	61.82−64.34	69.00	−5.92 *
	Sense of community at work	SW	66.46	65.21−67.71	77.00	−10.54 **
Work–individual interface	Job insecurity	JI	46.10	44.70−47.50	39.00	7.10 *
	Insecurity over working conditions	IW	52.54	51.22−53.86	41.00	11.54 **
	Quality of work	QW	65.06	63.83−66.30	71.00	−5.94 *
	Job satisfaction	JS	61.79	60.74−62.84	56.00	5.79 *
	Work-life conflict	WF	43.26	41.96−44.56	42.00	1.26
Social capital	Horizontal trust	TE	64.16	62.84−65.48	62.00	2.16
	Vertical trust	TM	62.39	61.23−63.55	64.00	−1.61
	Organizational justice	JU	61.23	60.06−62.41	57.00	4.23
Health and well-being	Self-rated health	GH	59.70	58.02−61.38	63.00	−3.30

^a^ Scale mean of the COPSOQ III of 23,361 employees in Canada, Spain, France, Germany, Sweden, and Turkey in 2016–2017. * Differences in the mean score of ≥|5 points| correspond to a small effect size (0.2–0.33). ** Differences in the mean score of ≥|10 points| correspond to an intermediate or high effect size (0.4–0.66). COPSOQ III means Copenhagen Psychosocial Questionnaire III.

**Table 3:** Comparison of psychosocial factors of Chinese health workers across four occupational statuses (N = 1054).

Domain	Dimension	Senior Hospital Manager (Mean ± SD)	Department Manager (Mean ± SD)	Department Staff (Mean ± SD)	Intern/ Trainee/Student (Mean ± SD)	*p* *
Demands at work	QD	53.99 ± 22.17	46.74 ± 17.58	35.68 ± 19.18	41.18 ± 23.99	<0.001
	WP	48.37 ± 25.09	62.83 ± 18.40	59.12 ± 21.78	52.58 ± 23.21	<0.001
	ED	50.00 ± 22.47	50.72 ± 17.47	41.98 ± 18.66	45.52 ± 21.00	<0.001
	HE	59.78 ± 20.05	59.20 ± 16.01	53.53 ± 20.32	52.07 ± 19.04	0.009
	subtotal	53.03 ± 17.95	54.87 ± 11.85	47.58 ± 15.50	47.84 ± 18.13	<0.001
Work organization and job contents	IN	56.25 ± 16.64	53.26 ± 14.91	43.73 ± 16.45	46.95 ± 19.86	<0.001
	PD	63.04 ± 22.45	67.61 ± 18.79	61.70 ± 18.76	60.81 ± 21.13	0.016
	CT	51.36 ± 17.37	37.93 ± 15.85	33.66 ± 17.30	42.87 ± 20.31	<0.001
	MW	72.28 ± 20.63	72.28 ± 23.40	73.49 ± 20.63	62.09 ± 25.51	<0.001
	subtotal	60.73 ± 15.34	57.77 ± 12.22	53.15 ± 12.01	53.18 ± 15.97	<0.001
Interpersonal relations and leadership	PR	60.87 ± 22.39	61.85 ± 18.94	56.82 ± 21.11	58.88 ± 21.86	0.080
	RE	66.30 ± 27.81	66.09 ± 23.69	65.72 ± 22.51	60.54 ± 25.36	0.136
	CL	69.20 ± 21.82	72.39 ± 18.91	69.96 ± 19.23	62.40 ± 21.28	<0.001
	CO	55.98 ± 18.80	48.48 ± 17.92	46.54 ± 19.40	53.20 ± 19.40	0.001
	IT	57.61 ± 27.63	44.35 ± 20.43	44.87 ± 23.33	51.65 ± 23.44	0.002
	QL	55.44 ± 25.20	62.90 ± 19.72	56.55 ± 20.33	58.75 ± 21.11	0.016
	SS	56.52 ± 27.40	61.41 ± 22.00	60.33 ± 22.25	57.95 ± 19.30	0.522
	SC	55.43 ± 25.51	63.70 ± 21.20	64.04 ± 20.32	57.64 ± 22.03	0.004
	SW	55.43 ± 27.91	64.24 ± 24.33	67.99 ± 19.54	60.64 ± 21.08	<0.001
	subtotal	59.20 ± 18.05	60.60 ± 13.19	59.20 ± 12.35	57.96 ± 14.75	0.480
Work–individual interface	JI	44.57 ± 26.87	39.13 ± 23.91	45.77 ± 23.01	55.17 ± 20.52	<0.001
	IW	43.84 ± 21.65	50.87 ± 18.65	52.38 ± 22.49	56.82 ± 19.81	0.029
	QW	48.91 ± 30.60	66.30 ± 20.95	66.23 ± 19.82	59.30 ± 19.40	<0.001
	JS	53.99 ± 20.55	64.71 ± 16.46	62.00 ± 17.28	59.09 ± 17.71	0.012
	WF	52.72 ± 17.66	50.11 ± 20.45	41.48 ± 21.67	46.69 ± 20.01	<0.001
	subtotal	48.80 ± 15.50	54.22 ± 10.78	53.57 ± 11.03	55.41 ± 12.27	0.059
Social capital	TE	56.52 ± 30.36	63.26 ± 21.79	64.78 ± 21.84	62.40 ± 19.93	0.219
	TM	55.07 ± 21.43	63.84 ± 18.20	62.62 ± 19.30	60.88 ± 19.21	0.184
	JU	53.26 ± 22.99	65.22 ± 19.01	60.79 ± 19.59	61.88 ± 17.44	0.026
	subtotal	54.95 ± 21.97	64.11 ± 17.86	62.73 ± 17.62	61.72 ± 15.80	0.136

* One-way ANOVA was used.

**Table 4:** Comparison of health-rated outcomes and turnover intention among Chinese health workers across occupational status (N = 1054).

Variable	Total	Senior Manager/ Hospital Manager	Department Manager	Department Staff	Intern/ Trainee/Student	*p*
Sleeping Troubles	38.15 ± 22.00	53.26 ± 24.92	37.28 ± 20.43	38.36 ± 22.14	34.66 ± 20.87	0.003 **
Burnout	37.14 ± 20.84	45.92 ± 23.43	39.73 ± 19.65	36.48 ± 21.00	37.40 ± 20.03	0.083
Stress	36.33 ± 20.17	51.09 ± 23.88	39.93 ± 19.54	35.45 ± 20.00	35.88 ± 19.95	0.001 ***
Depressive Symptoms	28.59 ± 20.48	37.77 ± 26.62	31.36 ± 18.45	27.78 ± 20.08	29.55 ± 23.03	0.041 *
General health	59.70 ± 27.82	57.61 ± 37.26	60.22 ± 29.04	58.65 ± 27.23	66.53 ± 27.87	0.035 *
Turnover Intention ^1^	116 (11.01%)	6 (0.57%)	15 (1.42%)	71 (6.74%)	24 (2.28%)	<0.001 ***

Data are Mean ± SD and n (%). * *p* < 0.05; ** *p* < 0.01; *** *p* < 0.001. ^1^ Pearson’s chi-square test of independence was used to compare turnover intention across career stages. One-way ANOVA was used in other groups of general health.

## Data Availability

The datasets used and analyzed in this study are available from the corresponding author upon reasonable request.

## Abbreviations

The following abbreviations are used in this manuscript:

WHO	The World Health Organization
HWs	Health Workers
ILO	The International Labour Organization
COPSOQ III	Copenhagen Psychosocial Questionnaire III
